# IFN-γ^+^ cytotoxic CD4^+^ T lymphocytes are involved in the pathogenesis of colitis induced by IL-23 and the food colorant Red 40

**DOI:** 10.1038/s41423-022-00864-3

**Published:** 2022-04-25

**Authors:** Lili Chen, Zhengxiang He, Bernardo S. Reis, Jesse D. Gelles, Jerry Edward Chipuk, Adrian T. Ting, Julie A. Spicer, Joseph A. Trapani, Glaucia C. Furtado, Sergio A. Lira

**Affiliations:** 1 Precision Immunology Institute, Icahn School of Medicine at Mount Sinai, New York, NY 10029 USA; 2 Laboratory of Mucosal Immunology, The Rockefeller University, New York, NY 10065 USA; 3 Department of Oncological Sciences, Icahn School of Medicine at Mount Sinai, New York, NY 10029 USA; 4 The Tisch Cancer Institute, Icahn School of Medicine at Mount Sinai, New York, NY 10029 USA; 5 The Diabetes, Obesity, and Metabolism Institute, Icahn School of Medicine at Mount Sinai, New York, NY 10029 USA; 6 Department of Immunology, Mayo Clinic, Rochester, MN 55905 USA; 7 Auckland Cancer Society Research Centre, Faculty of Medical and Health Sciences, The University of Auckland, Private Bag 92019, Auckland, 1142 New Zealand; 8 Cancer Immunology Program, Peter MacCallum Cancer Centre, 305 Grattan Street, Melbourne, VIC 3000 Australia

**Keywords:** Allura Red; IL23; Cytotoxic CD4^+^ T cells; CD4^+^ CTL; Inflammation; Epithelium damage; Colitis, Inflammation, Mucosal immunology

## Abstract

The food colorant Red 40 is an environmental risk factor for colitis development in mice with increased expression of interleukin (IL)-23. This immune response is mediated by CD4^+^ T cells, but mechanistic insights into how these CD4^+^ T cells trigger and perpetuate colitis have remained elusive. Here, using single-cell transcriptomic analysis, we found that several CD4^+^ T-cell subsets are present in the intestines of colitic mice, including an interferon (IFN)-γ-producing subset. In vivo challenge of primed mice with Red 40 promoted rapid activation of CD4^+^ T cells and caused marked intestinal epithelial cell (IEC) apoptosis that was attenuated by depletion of CD4^+^ cells and blockade of IFN-γ. Ex vivo experiments showed that intestinal CD4^+^ T cells from colitic mice directly promoted apoptosis of IECs and intestinal enteroids. CD4^+^ T cell-mediated cytotoxicity was contact-dependent and required FasL, which promoted caspase-dependent cell death in target IECs. Genetic ablation of IFN-γ constrained IL-23- and Red 40-induced colitis development, and blockade of IFN-γ inhibited epithelial cell death in vivo. These results advance the understanding of the mechanisms regulating colitis development caused by IL-23 and food colorants and identify IFN-γ^+^ cytotoxic CD4^+^ T cells as a new potential therapeutic target for colitis.

## Introduction

Intestinal CD4^+^ T cells are essential mediators of immune homeostasis and inflammation and are believed to be key players in the pathogenesis of inflammatory bowel disease (IBD) [1]. CD4^+^ T cells are usually described as helper cells (Th cells) and regulatory cells (Tregs). Multiple subsets of CD4^+^ Th cells (Th1, Th2, Th17, Th9, Th22, and T follicular helper, Tfh) in the intestine have been described [2]. Excessive activation of effector CD4^+^ T cells and/or reductions in CD4^+^ T cell-mediated tolerance to gut antigens are thought to play a role in the development of the two most common forms of IBD, ulcerative colitis (UC) and Crohn’s disease (CD) [1, 2].

Interleukin (IL)-23 is a cytokine that has been implicated in the pathogenesis of intestinal inflammation both in humans and in experimental mouse models [3]. IL-23 promotes the expansion and maintenance of Th17 cells, which secrete the proinflammatory cytokine IL-17 [4, 5]. It also acts on cells of the innate immune system and contributes to inflammatory cytokine production and intestinal inflammation [6]. Furthermore, IL-23 promotes natural killer (NK) cell activation and cytotoxicity in IBD [7]. Elevated production of IL-23 by myeloid cells has been noted in both patients with CD and patients with UC [8, 9], but expression of IL-23 by myeloid cells does not appear to be sufficient for induction of colitis in adult mice [10, 11], suggesting that other factors contribute to disease development.

We developed mice that overexpress IL-23 in CX3CR1-positive myeloid cells upon tamoxifen (TAM) treatment (*R23FR* mice, generated by crossing Rosa26-lox-STOP-lox-IL23 mice with CX3CR1^CreER^ mice) [11]. We have recently reported that the food colorant Red 40, also known as Allura Red AC, can act as an environmental risk factor to trigger IBD-like colitis in *R23FR* mice [12]. Colitis development depends on activated CD4^+^ T cells. Colitis-inducing CD4^+^ (memory) T cells are found in the mesenteric lymph nodes (mLNs) during remission and are able to trigger disease when transferred to lymphopenic mice (*Rag1*^−*/*−^), but only upon Red 40 treatment [12]. How these effector CD4^+^ T cells cause intestinal inflammation and marked destruction of the intestinal epithelium has yet to be defined. In principle, CD4^+^ T cells can produce cytokines with cytotoxic properties [13]; recruit and activate other cells, such as macrophages and neutrophils, in situ to promote cytotoxicity [14]; or have direct cytotoxic effects [15–18]. Indeed, CD4^+^ cytotoxic T lymphocytes (CD4^+^ CTLs or ThCTLs) have been reported to be present in intestinal biopsies of patients with CD compared with healthy controls [15–18] and in lymphopenic mice with colitis induced by adoptive transfer of naïve T cells (CD4^+^CD45RB^high^ cells) [19]. Other studies have demonstrated the existence of CD4^+^ CTLs in the intraepithelial lymphocyte (IEL) compartment in the small intestine [20, 21]. However, the ability of these CD4^+^ T cells to exert cytotoxic functions and the identities of their target cells are unresolved. Most evidence supporting the existence and functionality of these cells is indirect, based on the expression of surface markers or cytotoxicity-related molecules without assessment of direct cytolytic activity. Other studies have questioned the existence of CD4^+^ cytotoxic cells in the mucosa despite the expression of cytolytic molecules such as perforin and granzyme B [22]. Thus, the existence of functional cytotoxic CD4^+^ T cells in the mucosa and the contribution of these cells to colitis remain unresolved.

Here, we identified a population of intestinal IFN-γ-secreting CD4^+^ CTLs in experimental colitis induced by IL-23 and Red 40. This population displayed robust cytolytic capacity against intestinal epithelial cells (IECs) ex vivo and in vivo. Here, we report that IFN-γ^+^CD4^+^ T cells display direct ex vivo cytolytic activity and demonstrate that genetic ablation of IFN-γ or its blockade inhibits CD4^+^ CTL generation in vivo.

## Results

### Identification of cytotoxic CD4^+^ T cells by single-cell RNA sequencing

**Fig. 1:**
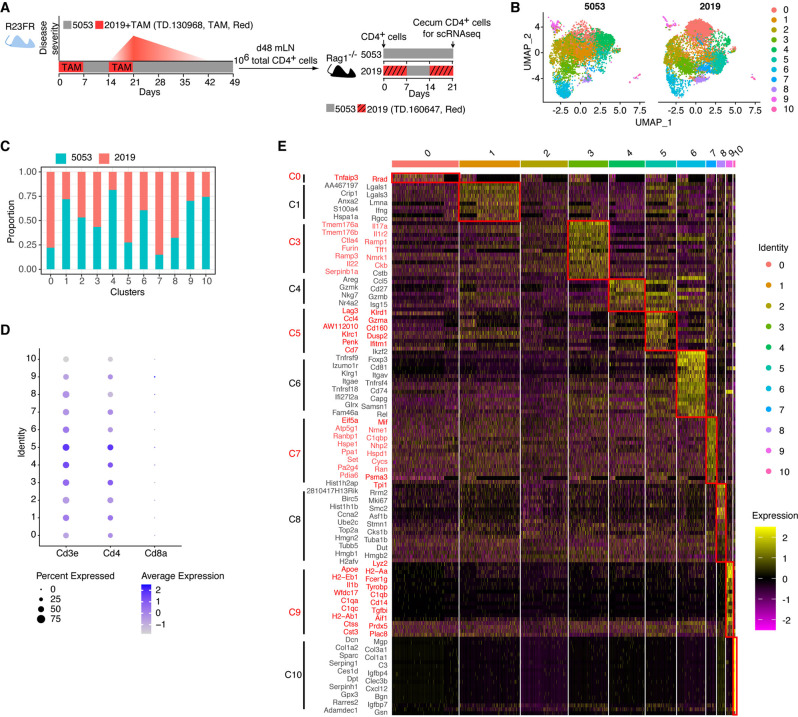
Single-cell RNA-seq analysis of cecal CD4^+^ T cells from adoptively transferred Rag1^−/−^ mice. **A** Colitis development in *R23FR* mice depends on CD4^+^ T cells. *R23FR* mice were treated with two 7-day cycles of tamoxifen (TAM) dissolved in Diet 2019 containing Red 40. *R23FR* mice showed colitis after the second cycle of TAM (Day 21). This colitis was transient, and the mice entered remission. Administration of Red 40 to these mice caused a flare of colitis (Day 56). Disease activity is shown in red. Total CD4^+^ T cells from the mLNs of *R23FR* mice in remission (Day 48) were enriched with microbeads and transferred into *Rag1*^−*/−*^ mice (10^6^ cells/mouse). Recipient *Rag1*^*−/−*^ mice were fed with our facility standard diet (Diet 5053) or with two alternating cycles of a Red 40-containing diet, TD.160647 (Diet 2019), for 21 days. Flow-sorted cecal CD4^+^ T cells from adoptively transferred *Rag1*^*−/−*^ mice at Day 21 were used for scRNA-seq analysis. **B** The UMAP plots showed distinct clustering of cecal CD4^+^ T cells isolated from adoptively transferred *Rag1*^*−/−*^ mice fed Diet 2019 or 5053. **C** Proportion of cells in each cluster according to the diet used. **D** Dot plot showing the *Cd3e*-, *Cd4*- and *Cd8a*-expressing cells from the different clusters. The size of the dot corresponds to the percentage of cells expressing the marker in each cluster. The color represents the average expression level. **E** Heatmap of differential gene expression in each cluster as assessed by scRNA-seq (*p* value < 0.05, average log_2_(fold change) >0.7, top 20). The color represents the expression level (log TPM)

CD4^+^ T cells obtained from the mLNs of *R23FR* mice in remission (primed by TAM-induced IL-23 and Red 40 treatment) were used to transfer colitis to *Rag1*^−*/*−^ mice treated with Red 40 in their drinking water (0.25 g/L) or diet (0.25 g/kg, TD.160647, diet 2019) (Fig. 1A) [12]. We refer to this CD4^+^ T-cell transfer model as the *R23FR* → *Rag* CD4^+^ T-cell transfer model. To better examine how disease develops in this setting, we performed a longitudinal histological and flow cytometric study (Fig. S1A). Flow cytometric analyses showed that the total numbers of CD45^+^ cells and CD4^+^ T cells in the cecum were not significantly different between adoptively transferred *Rag1*^−/−^ mice fed the Red 40-containing Diet 2019 and adoptively transferred *Rag1*^*−/*−^ mice fed Diet 5053 (without Red 40) on Day 14 (Fig. S1B–S1C). We found that the numbers of CD4^+^ T cells and CD45^+^ cells in the cecum increased over time (from Day 14 to Day 21) in adoptively transferred *Rag1*^*−/−*^ mice fed Diet 2019 but not in adoptively transferred *Rag1*^*−/−*^ mice fed Diet 5053 (Fig. S1B–S1C). Consistent with the flow cytometric results (Fig. S1B–S1C), there were no inflammatory infiltrates in the cecum in adoptively transferred *Rag1*^−*/−*^ mice fed Diet 2019 on Day 14 (Fig. S1D). However, by Day 16, we observed the presence of leukocytic infiltrates in the cecal mucosa without ulceration (Fig. S1D). By Day 18, we observed epithelial damage and ulceration in 3 of 5 mice (Fig. S1D), which progressed in all 5 mice to severe colitis with marked leukocytic infiltration, crypt loss, epithelial damage, and ulcerations by Day 21 (Fig. S1D). These data indicate that the food colorant Red 40 promotes progressive inflammation in the ceca of *Rag1*^*−/−*^ mice transferred with primed CD4^+^ T cells.

Although the Th17-cell subset is most prominently associated with IL-23R function, our recent work suggests that the immunopathology driven by Red 40 and IL-23 does not depend on classical Th17 responses, as blocking IL-17A and IL-17F fails to prevent colitis development in *R23FR* mice [12]. Therefore, we sought to further characterize the biology and function of these pathogenic CD4^+^ T cells. To do so, we performed single-cell RNA sequencing (scRNA-seq) on flow-sorted CD4^+^ T cells from the ceca of adoptively transferred *Rag1*^*−/−*^ mice at the end of treatment with Red 40 (Day 21) (Fig. 1A). Using uniform manifold approximation and projection (UMAP), we observed that CD4^+^ T cells obtained from mice treated with a Red 40-containing diet (Diet 2019) differed from those obtained from mice treated with Diet 5053 (without Red 40) (Fig. 1B). CD4^+^ T cells derived from the ceca of the transplanted *Rag1*^*−/−*^ mice formed 11 clusters (Fig. 1B). Our analysis also showed that the majority of cells present within Clusters 0, 5, 7 and 8 were derived from the ceca of Diet 2019-treated *Rag1*^*−/−*^ mice (Fig. 1C). In accordance with the sorting strategy (live CD45^+^CD3^+^CD4^+^CD8^-^ cells) (Fig. 1A), all cell subsets had high expression of *Cd3e* and *Cd4* but not *Cd8a* (Fig. 1D). Differential expression analyses of these clusters showed that the cells in all clusters were markedly different, although the number of differentially expressed genes varied among the clusters (Fig. 1E).

**Fig. 2:**
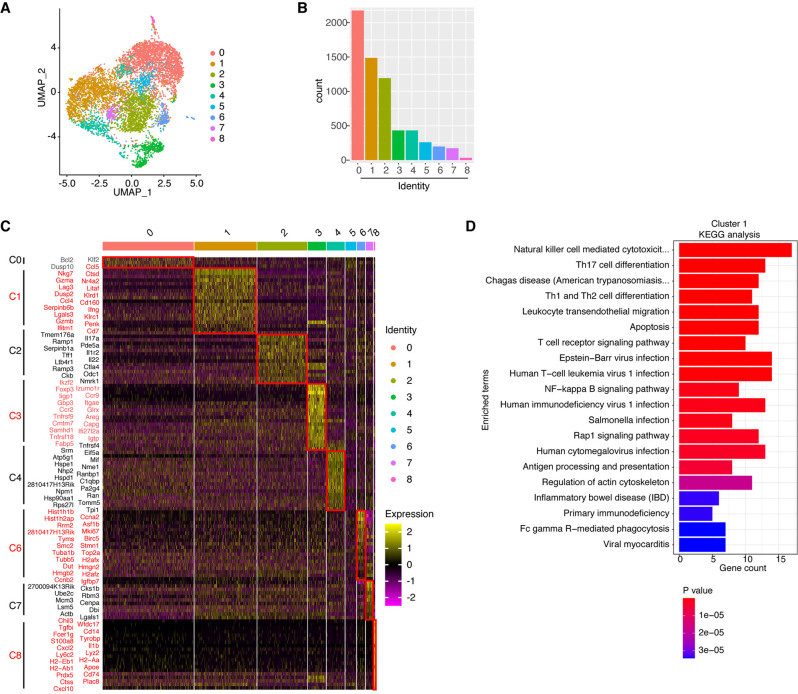
Single-cell RNA-seq analysis of colitogenic CD4^+^ T cells. **A** The UMAP plot shows distinct clustering of cecal CD4^+^ T cells isolated from adoptively transferred *Rag1*^*−/−*^ mice fed the Red 40-containing Diet 2019. **B** Cell counts for each cluster. **C** Heatmap of differential gene expression in each cluster as assessed by scRNA-seq (*p* value < 0.05, average log_2_(fold change) >0.6, top 20). The color represents the expression level (log TPM). **D** KEGG pathway enrichment analysis of upregulated genes in Cluster 1 (top 20 pathways)

Given that treatment with the Red 40-containing Diet 2019 induced activation of CD4^+^ T cells and was essential for disease development [12], we decided to focus our analyses on CD4^+^ T cells obtained from the ceca of colitic mice. Nine clusters were revealed by unbiased clustering of cecal CD4^+^ T cells from a pool of samples from 3 mice with colitis (6388 cells) (Fig. 2A). The predominant clusters were Clusters 0, 1, and 2 (Fig. 2B). Single-cell differential gene expression analysis among the nine clusters revealed notable differences in their molecular profiles (Fig. 2C). Differential expression analysis of the transcripts enriched in Cluster 1 showed significant overexpression of genes associated with cytotoxic function, including *Ifng*, *Gzma*, *Gzmb, Nkg7, Klrc1, Klrd1* and *Ccl4* (Fig. 2C). KEGG pathway enrichment analysis of the differentially expressed genes in Cluster 1 revealed that several canonical pathways associated with cytotoxic function, such as NK-cell-mediated cytotoxicity and FcγR-mediated phagocytosis, were upregulated in this cluster (Fig. 2D), which suggested that the cells in Cluster 1 had increased cytotoxic potential.

### CD4^+^ cells present in the intestines of primed mice rapidly promote intestinal epithelial apoptosis

**Fig. 3:**
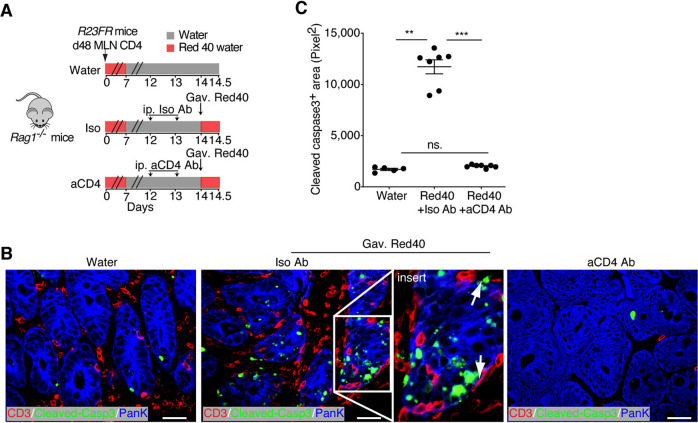
Intestinal CD4^+^ T cells cause epithelial damage in vivo. **A** Schematic representation of the experiment to investigate the numbers of apoptotic epithelial cells after anti-CD4 depletion. Anti-CD4 or an isotype antibody (0.2 mg) was administered daily for 2 days by intraperitoneal injection. Then, the mice were gavaged with 1 mg of Red40 and sacrificed after 12 h, and the large intestine was collected for histology. **B** Visualization of T cells (CD3^+^ cells) and apoptotic (Cleaved Caspase-3^+^) epithelial (pan-keratin^+^) cells in the ceca of isotype and anti-CD4 antibody-treated Red-40-gavaged adoptively transferred *Rag1*^*−/−*^mice. Scale bars = 25 μm. **C** Cleaved Caspase-3^+^ area in the ceca of anti-CD4 and isotype antibody-treated *Rag1*^*−/−*^ mice. *n* = 5–7. Each dot represents one mouse. Ns, not significant, **p < 0.01, ***p < 0.001, by nonparametric Mann–Whitney test

To investigate whether CD4^+^ T cells present in the intestine could promote epithelial cell death, we used the *R23FR* → *Rag* model. CD4^+^ T cells taken from the mLNs of primed mice were transferred to *Rag1*^*−/−*^ mice, and the mice were treated with Red 40 (0.25 g/L drinking water) for 1 week (Fig. 3A). Seven days later (Day 14), as expected, CD4^+^ T cells were present in the intestines of *Rag1*^*−/−*^ mice (Fig. 3B and Fig. S2). On Day 14, we gavaged these animals with Red 40 (1 mg/mouse) or water and examined their ceca 12 h later (Fig. 3A). We found marked increases in the numbers of apoptotic epithelial cells (Cleaved Caspase-3^+^pan-keratin^+^) in the ceca of Red-40-gavaged adoptively transferred *Rag1*^*−/−*^ mice compared with those in the ceca of control mice gavaged with water (Fig. 3B, C). Of note, T cells were abundant in the areas in close proximity to the colonic epithelial cells positively stained with Cleaved Caspase-3 in the Red-40-gavaged *Rag1*^*−/−*^ mice (Fig. 3B). Finally, we tested whether CD4^+^ T cells had a critical role in inducing epithelial cell death by depleting them shortly before Red 40 gavage (Fig. 3A). We observed that T cell depletion (Fig. S2A-S2C) significantly decreased the numbers of apoptotic epithelial cells (Cleaved Caspase-3^+^pan-keratin^+^) in the ceca of Red-40-gavaged mice compared with the numbers in isotype-treated controls (Fig. 3B, C). These results suggest that CD4^+^ T cells present in the intestines of primed mice promote epithelial damage.

### Pathogenic CD4^+^ T cells from colitic mice are cytotoxic to colonic epithelial cells ex vivo

**Fig. 4:**
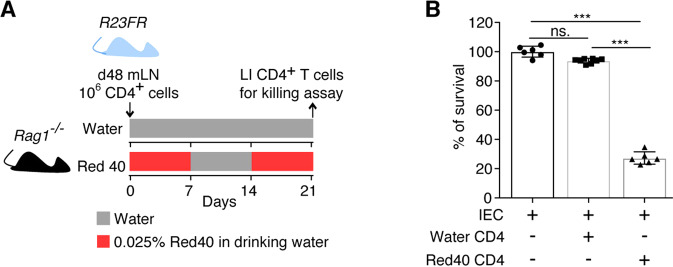
CD4^+^ T cells from the large intestines of adoptively transferred *Rag1*^*−/−*^ mice with colitis kill IECs ex vivo. **A** Schematic representation of the experiment. *R23FR* mLN CD4^+^ T cells were adoptively transferred into *Rag1*^*−/−*^ mice, and the mice were fed with or without 0.25 g/L Red 40 in drinking water. Flow-sorted CD4^+^ T cells from the large intestines of *Rag1*^*−/−*^ mice at Day 21 were used as effector cells. **B** Survival of target epithelial cells after coculture with CD4^+^ T cells was assessed with a CellTiter-Glo Luminescent Cell Viability Assay Kit (effector:target ratio = 4:1). *n* = 6. Each dot represents one well/condition from a representative of three independent experiments with similar results. Ns, not significant, ***p < 0.001, by nonparametric Mann–Whitney test

Having shown that CD4^+^ T cells from adoptively transferred *Rag1*^*−/−*^ mice could promote the killing of IECs in vivo, we next tested whether these intestinal CD4^+^ T cells could directly kill IECs. To do so, we isolated DAPI^−^CD45^+^CD3^+^CD4^+^CD8^−^ cells from the ceca of adoptively transferred *Rag1*^*−/−*^mice with colitis (Day 21) and from control adoptively transferred *Rag1*^*−/−*^ mice without disease (Day 21), cocultured them with C57BL/6 mouse primary IECs (Cell Biologics, Chicago, IL) and examined cell survival using a CellTiter-Glo Luminescent Cell Viability Assay Kit (Fig. 4A). We found that intestinal CD4^+^ T cells from adoptively transferred *Rag1*^*−/−*^ mice treated with Red 40 were cytotoxic against epithelial cells (Fig. 4B). No cytotoxicity was observed when intestinal CD4^+^ T cells from mice treated with water were used (Fig. 4B).

**Fig. 5:**
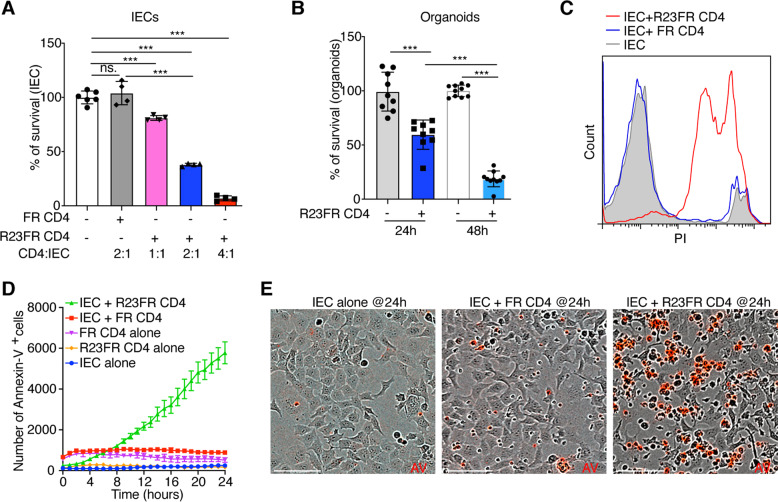
Intestinal CD4^+^ T cells from R23FR mice with colitis kill IECs ex vivo. CD4^+^ T cells obtained from the large intestines of *R23FR* mice and *FR* mice at Day 56 were used for ex vivo cytotoxicity assays. **A** Relative survival of target epithelial cells after coculture with CD4^+^ T cells from *R23FR* and *FR* mice for 24 h as determined using a CellTiter-Glo Luminescent Cell Viability Assay Kit with different effector:target ratios. *n* = 4–6. Each dot represents one well/condition from a representative of four independent experiments with similar results. **B** Relative survival of target intestinal enteroids after coculture with CD4^+^ T cells from *R23FR* mice for 24 h and 48 h as determined using a CellTiter-Glo Luminescent Cell Viability Assay Kit (effector:target ratio = 4:1). *n* = 9–10. Each dot represents one well/condition from a representative of three independent experiments with similar results. **C** Flow cytometric analysis of the number of dead epithelial (PI^+^Epcam^+^) cells after culture of IECs with colonic CD4^+^ T cells from *R23FR* or *FR* mice at Day 56 (effector:target ratio = 4:1) for 24 h. The data represent two independent experiments. **D** IncuCyte kinetic quantification of apoptotic (Annexin V^+^) cells under different conditions. IEC alone, CD4^+^ T cells from *R23FR* or *FR* mice alone and IECs cultured with CD4^+^ T cells from *R23FR* or *FR* mice (effector:target ratio = 2:1). The data are expressed as the average of the events per well (*n* = 6 wells/condition). **E** Representative IncuCyte images of Annexin V^+^ cells (AV, red) showing IECs alone and IECs cocultured with colonic CD4^+^ T cells from control *FR* mice or from *R23FR* mice. Ns, not significant, ***p < 0.001, by nonparametric Mann–Whitney test. Scale bars = 100 μm in (**E**)

As the CD4^+^ T cells in *Rag1*^*−/−*^ recipients originated from a *R23FR* donor in remission (Fig. 4A), we next tested whether CD4^+^ T cells of *R23FR* mice with colitis could directly promote the killing of IECs. We isolated DAPI^−^CD45^+^CD3^+^CD4^+^CD8^−^ cells from the ceca of *R23FR* mice with colitis (Day 56) and from control *FR* mice [11, 12] (Day 56), cocultured them with IECs, and measured cell survival using a CellTiter-Glo Luminescent Cell Viability Assay Kit. We observed a marked decrease in viable IECs cocultured with CD4^+^ T cells isolated from *R23FR* mice compared to those exposed to control *FR* intestinal CD4^+^ T cells (Fig. 5A). The cytotoxicity of the CD4^+^ T cells toward epithelial cells was cell number-dependent (Fig. 5A). Furthermore, we observed that CD4^+^ T cells from the ceca of *R23FR* mice with colitis (Day 56) lysed colonic epithelial cell enteroids from wild-type (WT) mice with an efficiency similar to that observed using primary epithelial cells as targets (Fig. 5B).

To further validate that CD4^+^ T cells of *R23FR* mice with disease could directly promote the killing of IECs, we performed additional ex vivo cytotoxicity assays. We examined cytotoxicity using flow cytometry and confirmed that CD4^+^ T cells isolated from *R23FR* mice with colitis had potent killing ability and that the majority of the dead cells were epithelial cells, as indicated by propidium iodide (PI)-positive costaining with the epithelial cell marker Epcam (Fig. 5C). In addition, by using the IncuCyte platform [23, 24], we found that the numbers of apoptotic (Annexin V^+^) cells were significantly higher in wells containing IECs cocultured with CD4^+^ T cells from *R23FR* mice with colitis than in wells containing IECs alone or IECs cocultured with control *FR* intestinal CD4^+^ T cells (Fig. 5D, E). Consistent with these findings, there was a significant increase in intestinal permeability in *R23FR* mice relative to controls (*FR* mice) assessed 5 h after administration of FITC-dextran (Fig. S3).

Our recent work shows that Red 40 treatment of WT mice with systemic overexpression of IL-23 results in the development of colitis [12]. To determine if intestinal CD4^+^ T cells in this model can directly kill IECs ex vivo, we isolated DAPI^−^CD45^+^CD3^+^CD4^+^CD8^−^ cells from the large intestines of IL-23 minicircle DNA-injected WT mice treated with or without Red 40 (Day 21), cocultured them with IECs, and measured cell survival using a CellTiter-Glo Luminescent Cell Viability Assay Kit (Fig. S4A–S4B). Consistent with the histological findings [12], intestinal CD4^+^ T cells from mice treated with Red 40 were cytotoxic against epithelial cells, but no cytotoxicity was observed when intestinal CD4^+^ T cells from mice treated with water were used (Fig. S4C).

Together, these results indicate that intestinal CD4^+^ T cells from colitic mice treated with Red 40 are cytotoxic to colonic epithelial cells, suggesting that CD4^+^ CTLs are involved in the pathogenesis.

### CD4^+^ T cell-mediated cytotoxicity is contact dependent and involves the FasL pathway

**Fig. 6:**
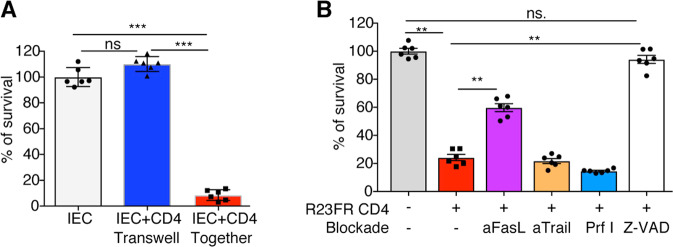
Contributions of perforin, FasL and TRAIL to CD4^+^ T cell-mediated cytotoxicity. Relative survival of target epithelial cells after 24 h of coculture with colonic CD4^+^ T cells from Day-56 colitic *R23FR* mice under different conditions as determined using a CellTiter-Glo Luminescent Cell Viability Assay Kit. **A** CD4^+^ T-cell cytotoxicity requires cell-to-cell contact. Epithelial cells were seeded in a transwell chamber bottom receiver plate. Effector cells (CD4^+^ T cells) were plated in the top chamber insert (effector:target ratio = 4:1). The transwell chamber was incubated at 37 °C for 24 h, and cell survival was measured as described above. **B** Relative survival of target epithelial cells after coculture with CD4^+^ T cells in the presence of antibodies (anti-FasL and anti-Trail) or pharmacological agents (SN34960 and z-VAD) (effector:target ratio = 2:1). SN34960 is a specific perforin inhibitor (Prf I), and z-VAD is a pan-caspase inhibitor. *n* = 6. Each dot represents one well/condition from a representative of four independent experiments with similar results. ns, not significant, **p < 0.01, ***p < 0.001, by nonparametric Mann–Whitney test

We hypothesized that CD4^+^ CTL-mediated cytotoxicity against IECs could occur through secretion of soluble factors or require direct cell–cell contact. To test these hypotheses, we used a transwell chamber assay and found that cytotoxicity was abolished when CD4^+^ T cells and IECs were kept separated (Fig. 6A), suggesting that CD4^+^ CTL-mediated cytotoxicity against IECs required cell-to-cell contact. CD4^+^ CTLs can kill target cells in a cell-to-cell contact manner via surface expression of TNF family members, including Fas ligand (FasL or CD95L) and TNF-related apoptosis-inducing ligand (TRAIL), or via the perforin/granzyme-mediated cytotoxic pathway [25]. We thus investigated the expression of *Fasl*, *Tnfsf10* (encoding TRAIL), *Prf1* (encoding perforin), *Gzma*, and *Gzmb* in the CD4^+^ T-cell subsets identified by scRNA-seq (Fig. S5). In Cluster 1, there was increased expression of *Ifng*, *FasL*, *Gzma* and *Tnfsf10* (Fig. S5)*. Gzmb* expression was also found in this cluster but was the highest in Cluster 3 (Fig. S5). *Prf1* expression was extremely low in all clusters (Fig. S5). We next examined the contributions of these factors to CD4^+^ cytotoxicity against IECs. Granzymes are necessary for triggering apoptosis of target cells but require perforin to be appropriately delivered into the target cell cytosol [26]. To determine whether a perforin-dependent mechanism was responsible for the cytotoxicity induced by CD4^+^ T cells, we pretreated effector cells with SN34960, a small molecule inhibitor of perforin [27]. We found that the inhibition of perforin did not inhibit CD4^+^ CTL-mediated cytotoxicity (Fig. 6B), consistent with the lack of *Prf1* expression in these CD4^+^ T cells (Fig. S5). No inhibition of cytotoxicity was observed when anti-TRAIL antibodies were used (Fig. 6B). Notably, the use of an anti-FasL antibody partially inhibited CD4^+^ CTL-mediated cytotoxicity against epithelial cells (Fig. 6B). The CD4^+^ CTL-mediated cytotoxicity against IECs was almost completely prevented by the poly-caspase inhibitor z-VAD-fmk (Fig. 6B), indicating that the death of IECs was caspase dependent. These results indicate that CD4^+^ T cell-mediated cytotoxicity involves triggering of caspase activity in the target IECs that is mostly mediated by FasL but not TNF-α, IFN-γ, TRAIL, or perforin.

### IFN-γ^+^CD4^+^ T cells have a potent killing ability

**Fig. 7:**
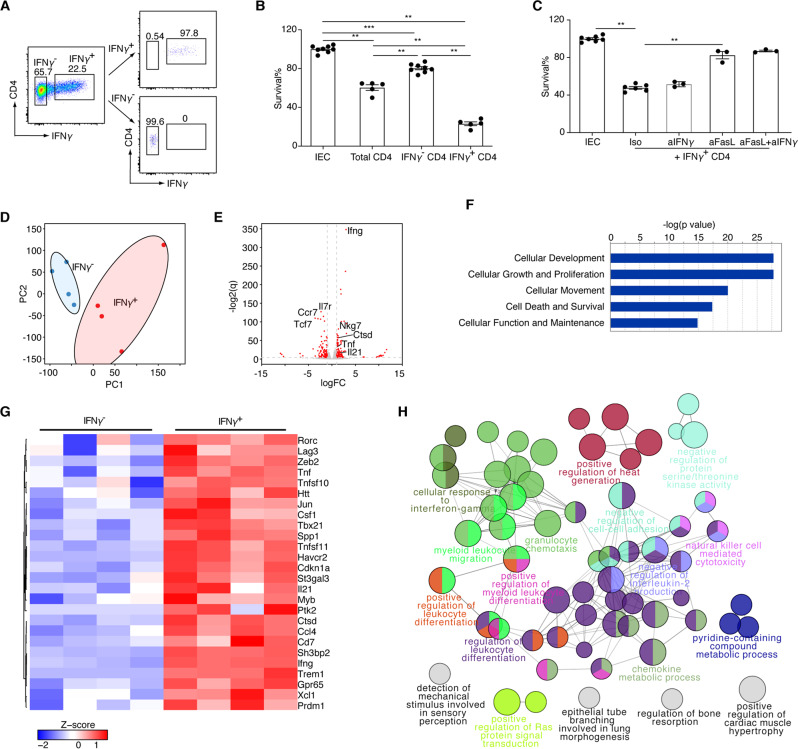
IFN-γ^+^CD4^+^ T cells exhibit elevated cytolytic activity. **A** Sorting strategy of intestinal IFN-γ^+^ and IFN-γ^-^ CD4^+^ T cells. **B** Relative survival of target epithelial cells after coculture with IFN-γ^+^ and IFN-γ^-^ CD4^+^ T cells from colitic *R23FR* mice for 24 h as assessed using a CellTiter-Glo Luminescent Cell Viability Assay Kit (effector:target ratio = 2:1). Each dot represents one well/condition from a representative of three independent experiments with similar results. *n* = 5–8, **p < 0.01, ***p < 0.001, by nonparametric Mann–Whitney test. **C** Relative survival of target epithelial cells after coculture with IFN-γ^+^CD4^+^ T cells in the presence of anti-FasL and anti-IFN-γ (effector:target ratio = 4:1). Each dot represents one well/condition from a representative of two independent experiments with similar results. *n* = 3–7, **p < 0.01, by nonparametric Mann–Whitney test. **D** Principal component analysis of RNA-seq expression data from cecal IFN-γ^+^ and IFN-γ^−^ CD4^+^ T cells from colitic *R23FR* mice (*n* = 4/group). **E** Plot of the log 2 q (log 2 FDR-adjusted *P* value) vs. log 2 FC (log 2(fold change)) of all detected transcripts. The points are colored according to expression status: nonsignificant genes (gray) and significant genes (*Q* < 0.05; log 2 FC > 1 or log 2 FC <  −1; red). **F** The top 5 functional annotations in the Biological Function category were determined for genes significantly changed between IFN-γ^+^ cells and IFN-γ^−^ cells by using Ingenuity Pathway Analysis (IPA) software. **G** Heatmap showing upregulated genes in IFN-γ^+^ cells associated with cell death and survival pathways. **H** ClueGo enrichment analysis of Gene Ontology Biological Process terms for the significantly upregulated genes in IFN-γ^+^ cells. The nodes represent significantly enriched GO term groups, including “natural killer cell-mediated cytotoxicity”. The node size represents the term enrichment significance. Differently grouped terms are shown in different colors. Functionally related groups partially overlap

As shown in the scRNA-seq analysis, we found that cells in Cluster 1 expressed markers associated with cytotoxic function, including *Ifng*, *Nkg7, Gzma*, *Gzmb* and *Fasl* (Fig. S5). We hypothesized that IFN-γ^+^CD4^+^ T cells could be a prominent cytotoxic subset in the gut. To test this hypothesis, we isolated IFN-γ^+^ and IFN-γ^-^ cells with microbeads from *R23FR* mice with disease (Fig. 7A) and performed ex vivo cytotoxic assays (Fig. 7B). We found that IFN-γ^+^CD4^+^ T cells had a more potent killing ability than IFN-γ^-^CD4^+^ T cells (Fig. 7B). Consistent with the results shown above, we found that the use of an anti-FasL antibody inhibited IFN-γ^+^CD4^+^ T cell-mediated cytotoxicity against epithelial cells ex vivo (Fig. 7C). Notably, the use of anti-IFN-γ did not block the cytotoxicity (Fig. 7C), indicating that IFN-γ could not account for the direct cytotoxicity elicited by CD4^+^ T cells against IECs.

To study the transcriptional profile accounting for a possible functional difference between IFN-γ^+^CD4^+^ and IFN-γ^−^CD4^+^ T cells, we sorted them from the ceca of colitic *R23FR* mice and subjected them to RNA sequencing and gene expression analysis (Fig. 7D–H). As shown in Fig. 7D, the transcriptome of IFN-γ^+^CD4^+^ cells differed significantly from that of IFN-γ^−^CD4^+^ cells. Differential expression analysis revealed 141 and 122 genes that were significantly upregulated and downregulated in cecal IFN-γ^+^ and IFN-γ^−^ CD4^+^ T cells, respectively (Fig. 7E). In accordance with the sorting strategy (Fig. 7A), *Ifng* was among the most differentially expressed genes (Fig. 7E). Notably, the transcript levels of genes including *Tnf*, *Il21*, and *Nkg7* were significantly higher in IFN-γ^+^CD4^+^ T cells than in their IFN-γ^−^ counterparts (Fig. 7E). To understand the relationships between the differentially expressed genes and physiological functions, we utilized Ingenuity Pathway Analysis (IPA, Ingenuity Systems, Inc.). The IPA results revealed that the cell death and survival pathway, one of the top 5 pathways, was enriched for the differentially expressed genes (Fig. 7F). Within the cell death and survival pathway, we found elevated expression of genes related to cytotoxicity (for example, *Tnf*, *Tnfsf10*, *Htt*, *Jun*, *Tbx21*, *Havcr2*, *Il21*, *Ifng* and *Xcl1*) in IFN-γ^+^CD4^+^ T cells (Fig. 7G). We confirmed this finding via ClueGo analysis of significantly upregulated genes in IFN-γ^+^CD4^+^ T cells, which identified a NK cell-mediated cytotoxic pathway (Fig. 7H). Together, these results indicate that IFN-γ^+^CD4^+^ T cells express cytotoxic genes at elevated levels, which confers on them a potent killing ability.

### IFN-γ is required for CD4^+^ CTL generation and activation in vivo

**Fig. 8:**
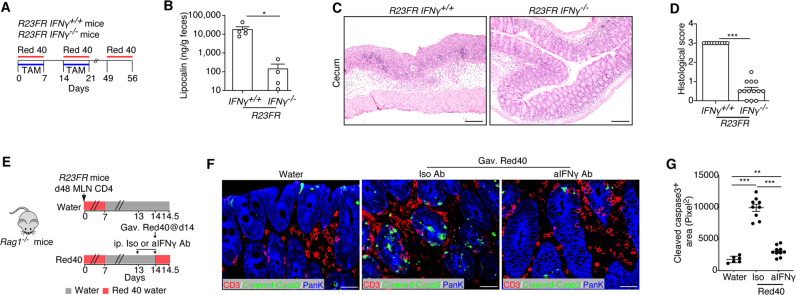
IFN-γ is required for CD4^+^ CTL generation and activation in vivo. **A** Schematic representation of the experiment to test the role of IFN-γ in CD4^+^ CTL generation. **B** Fecal lipocalin-2 levels of *R23FR/Ifng*^*-/-*^ mice and *R23FR/Ifng*^*+/+*^ mice after TAM + Red 40 treatment (Day 56). *n* = 4–5 mice/group. **C** and **D** Representative H&E-stained sections (**C**) and histological scores (**D**) of the ceca of *R23FR/Ifng*^−*/−*^ mice and *R23FR/Ifng*^*+/+*^ mice after TAM + Red 40 treatment (Day 56). *n* = 10–13 mice/group. Scale bars = 100 μm. **E** Schematic representation of the experiment to investigate the numbers of apoptotic epithelial cells after anti-IFN-γ blockade. One milligram of anti-IFN-γ or isotype antibody was administered by intraperitoneal injection prior to Red 40 gavage. **F** Visualization of T cells (CD3^+^ cells) and apoptotic (Cleaved Caspase-3^+^) epithelial (pan-keratin^+^) cells in the ceca of anti-IFN-γ-treated Red-40-gavaged adoptively transferred *Rag1*^−*/−*^ mice. Scale bars = 25 μm. **G** Cleaved Caspase-3^+^ areas in the ceca of anti-IFN-γ and isotype antibody-treated *Rag1*^*−/−*^ mice. *n* = 6–10 mice/group. Each dot represents one mouse. *p < 0.05, **p < 0.01, ***p < 0.001, by nonparametric Mann–Whitney test

CD4^+^ CTLs can develop from Th0, Th1, Th2, Th17, and Treg effector subsets [28]. However, CD4^+^ CTLs derived from Th1 (or Th1-like) cells represent the majority of CD4^+^ CTLs, which produce IFN-γ alone or together with other cytokines [29–31]. It is well known that the transcription factor T-bet functions as the master regulator of type 1 inflammatory responses and induces IFN-γ production. Indeed, compared with IFN-γ^-^CD4^+^ T cells, IFN-γ^+^CD4^+^ T cells had significantly higher expression of *Tbx21* (encoding T-bet) (Fig. 7G), suggesting that T-bet may promote CD4^+^ CTL differentiation [32]. Therefore, we hypothesized that IFN-γ might be required for CD4^+^ CTL generation contributing to colitis development in *R23FR* mice and *R23FR* → *Rag* transfer mice. We intercrossed *R23FR* mice with *Ifng*^−*/*−^ mice and treated their offspring with TAM + Red 40 (Fig. 8A). At the end of treatment (Day 56), *R23FR/Ifng*^*-/-*^ mice had much less severe colitis than *R23FR/Ifng*^*+/+*^ mice, as measured by histology and elevation of fecal lipocalin (Fig. 8B–D). In addition, we confirmed by immunostaining that T cells that were in close proximity to the cleaved Caspase-3^+^ epithelial cells were T-bet^+^ in the Red-40-gavaged *Rag1*^*−/−*^ mice (Fig. S6). Next, we examined whether blockade of IFN-γ could prevent CD4^+^ CTL activation, as indicated by increased numbers of apoptotic epithelial cells in vivo. We found that blockade of IFN-γ (Fig. 8E) significantly decreased the number of apoptotic epithelial cells (Cleaved Caspase-3^+^pan-keratin^+^) in the cecum in Red-40-gavaged adoptively transferred *Rag1*^*−/*−^ mice compared with isotype-treated controls (Fig. 8E–G). Together, these results suggest that IFN-γ is required for CD4^+^ CTL generation and activation in vivo.

## Discussion

Essential roles of CD4^+^ T cells have been demonstrated in several animal models of experimental colitis, most notably in the adoptive transfer of naïve T cells into lymphopenic hosts [33]. CD4^+^ T lymphocytes are also believed to play a key role in the pathogenesis of human IBD. Historically, it was believed that CD was driven by Th1 cells, while UC was thought to be mediated by Th2 cells. However, as new subsets of T cells have been identified (e.g., the Th17, Th22, Th9, and Tfh subsets), this paradigm has been revised. It has also been appreciated that CD4^+^ T cells can display plastic programs, converting from one subset into another. For instance, it has been reported that Th17 precursors can give rise to Th1-like cells [34] and that Tregs can convert into pathogenic T cells [35]. Here, we identify a pathogenic role for a new subset of IFN-γ-secreting CD4^+^ T cells in mice conditionally expressing IL-23 and exposed to a dietary factor (a food colorant) and show that these cells may contribute to intestinal epithelial damage.

IL-23 plays an important role in both innate and adaptive immune-driven colitis [36]. IL-23-responsive Group 3 innate lymphoid cells are the key population driving innate intestinal pathology [6]. Loss-of-function studies have suggested that IL-17^+^ and/or IL-17^+^IFN-γ^+^ CD4^+^ T cells are the major inducers of adaptive immune-driven colitis in a mouse model [34, 36]. Here, we show that IL-23 expression leads to the development of colitis upon Red 40 administration and that cytotoxic CD4^+^ T cells are the major population driving disease development. It has been reported that IL-23 plays a role in the long-term establishment of memory CD4^+^ T cells in the early phase of the immune response and induces strengthened sustained CD8^+^ CTL during viral infection [37]. We hypothesize that IL-23 might act on Red 40-specific memory CD4^+^ T cells generated during the priming process and that Red 40 re-exposure might cause activation of memory CD4^+^ CTL cells, contributing to intestinal epithelial damage. These findings may have implications for understanding the role of IL-23 in the CD4^+^ T cell-mediated immunopathogenesis of IBD.

The majority of the studies on CD4^+^ CTLs to date have focused on the protective roles of these cells in infectious diseases. CD4^+^ CTLs have been detected in various infectious conditions in both humans and experimental animals [38]. Other studies have suggested that CD4^+^ CTLs contribute to the progression of autoimmunity [39] and play a role in antitumor immunity [40, 41]. In the context of intestinal inflammation, CD4^+^ CTLs are enriched in intestinal biopsies of patients with IBD compared with those of healthy controls [15–18] and in mice with colitis induced by naïve T cells [19, 42, 43], but the related studies have mostly relied on analyses of the expression of phenotypic surface markers. In our study, we identified phenotypic markers on cytotoxic CD4^+^ T lymphocytes by scRNA-seq in the large intestines of adoptively transferred *Rag1*^*−/−*^ mice and later functionally proved this cytotoxic activity in vitro and in vivo.

Signals mediated by T-cell receptors, cytokines, costimulatory molecules, and other cell surface receptors are integrated and interpreted by a network of transcriptional regulators, which collectively orchestrate the differentiation of cytotoxic CD4^+^ T cells. The cellular and environmental factors that mediate the generation of intestinal CD4^+^ CTLs are incompletely defined, but it has been reported that upregulation of the transcription factor Runx3 in the absence of the master regulator ThPOK favors the development of this population in the small intestine [20, 44]. Similar to the CD4^+^ CTLs in the small intestine, the large intestine CD4^+^ CTLs described here expresses Runx3 but little, if any, ThPOK (encoded by *Zbtb7b*). Cytotoxic CD4^+^ T cells in the small intestine are believed to be transcriptionally reprogrammed from CD8^+^ T cells and characterized by the expression of CD8αα [20, 44]. Different from the CD4^+^ cytotoxic T cells present in the small intestine, the colonic CD4^+^ cytotoxic T cells identified here did not express *Cd8a*. Another transcription factor related to cytotoxicity function, Eomes, was not significantly expressed in any cluster. However, our scRNA-seq results showed that most CD4^+^ T cells expressing cytotoxic markers (Cluster 0) expressed *Tbx21*, which encodes the transcription factor T-bet. It has been reported that T-bet is important for regulation of IEL functional maturation [45]. We suggest that T-bet may have a similar functional role in the CD4^+^ cytotoxic T cells identified here. Consistent with the critical role of T-bet in the promotion of CD4^+^ CTL differentiation [32], we show here that IFN-γ is required for CD4^+^ CTL generation and activation in *R23FR* mice.

IFN-γ is one of the critical mediators of inflammatory disorders and one of the most abundant proinflammatory cytokines produced by mucosal CD4^+^ T cells in IBD patients. The expression of IFN-γ has been historically linked to CD4 Th1 populations, but it is now documented that Th17 and Treg cells can also produce IFN-γ. IFN-γ contributes to epithelial barrier defects in the gut by disassembling tight junctions, reducing the rate of IEC migration, and regulating IEC proliferation and apoptosis [46, 47]. IFN-γ, in synergy with TNF-α, inhibits IEC proliferation and promotes apoptosis to sustain intestinal inflammation by inhibiting the Wnt-β-catenin signaling pathway [46] to contribute to the pathology of IBD. Here, our results suggest that colitis triggered by IL-23 expression and Red 40 is dependent on IFN-γ and that IFN-γ is required for CD4^+^ CTL generation in *R23FR* mice. In addition, IFN-γ^+^ CD4^+^ T cells display highly ex vivo cytolytic activity. IFN-γ has a clear role in cytotoxicity induced by CD8^+^ T cells [48, 49]; therefore, one could speculate that it may also affect the expression of cytotoxicity-related molecules by cytotoxic CD4^+^ T cells in vivo. However, the results of our IFN-γ–blocking ex vivo experiments suggest that IFN-γ is not key to cytolytic activity. Thus, we suggest that IFN-γ may act directly on the development of CD4^+^ cytotoxic T cells in vivo or indirectly on other immune cells, such as APCs, which in turn could induce/activate CD4^+^ CTLs in vivo.

Studies testing the efficacy of the anti-IFN-γ antibody fontolizumab have shown that it is only slightly superior to placebo in inducing clinical remission in patients with acute CD [50–52]. These studies suggest that IFN-γ does not have a dominant role in the pathogenesis of CD, but they do not rule out the possibility that IFN-γ is important during disease inception or that it plays a role in IL-23-driven colitis in humans. Furthermore, the role of IFN-γ in the pathogenesis of UC remains unexplored. IFN-γ has been shown to have a role in the development of animal models of UC [53], but its role in UC in humans remains untested. Therefore, further clinical trials are needed to determine the efficacy of anti-IFN-γ therapy for UC.

It has been suggested that perforin/granzyme- and FasL-mediated cytotoxicity contribute to tissue injury in IBD [43, 54]. Exocytosis of perforin and granzymes from cytoplasmic vesicles appears to account for most of the observed cytotoxic activity of CD8^+^ T cells, whereas FasL/Fas-mediated cytolysis is a more common effector mechanism in cytotoxic CD4^+^ T cells [42, 55]. Consistent with this finding, we found that CD4^+^ CTL-mediated cytotoxicity against IECs was dependent on FasL but not TRAIL or perforin. Indeed, the kinetics of epithelial cell death were relatively slow, and cell death was not inhibited by a perforin inhibitor, suggesting that a perforin-dependent mechanism was unlikely. However, anti-FasL treatment only partially inhibited CD4^+^ CTL-mediated cytotoxicity, indicating either that an additional subsidiary pathway could also mediate cell death or that FasL blockade was not complete in our ex vivo killing assay. The fact that the CD4^+^ CTL-mediated cytotoxicity against epithelial cells was almost completely prevented by the poly-caspase inhibitor z-VAD-fmk indicates that any potential cell death mediators in addition to FasL must also operate via caspases.

Collectively, our studies uncover a previously unappreciated pathogenic role of a subset of IFN-γ-producing cytotoxic CD4^+^ T cells in IL-23-driven colitis. Our results add to the clinical evidence that targeting IL-23 is a useful and effective approach for treating patients with IBD and suggest that inhibition of IFN-γ could prevent the generation of CD4^+^ CTLs and inhibit disease.

## Materials and methods

### Study design

The food colorant Red 40 can act as an environmental risk factor to trigger IBD-like colitis in IL-23-overexpressing mice (*R23FR* mice) [12]. Colitis development depends on activated CD4^+^ T cells. Colitis-inducing CD4^+^ T cells are found in mLNs during remission and are able to trigger disease when transferred to *Rag1*^*−/−*^ mice, but only upon Red 40 treatment [12]. The goal of this study was to explore how these effector CD4^+^ T cells cause intestinal inflammation and marked destruction of the intestinal epithelium. CD4^+^ T cells isolated from the ceca of adoptively transferred *Rag1*^*−/−*^ mice were assessed by scRNA-seq. We investigated how CD4^+^ T cells killed epithelial cells in vivo. Ex vivo functional analyses were used to evaluate the cytotoxicity of these CD4^+^ T cells against IECs. In addition, we used genetic ablation to eliminate IFN-γ in order to assess its contribution to the generation of CD4^+^ CTLs and the induction of colitis. The sample size is specified in each figure legend, and the numbers of ex vivo experimental replicates are indicated in the figure legends.

### Mice

C57BL/6 J (stock #000664), *Rag1*^*−/−*^ (stock #002216) and *Ifng*^*−/−*^ (stock #002287) mice were purchased from The Jackson Laboratory (Bar Harbor, ME). The *R23FR* mice [11] and *FR* mice [11] have been described previously. All mice were on a C57BL/6 background. The mice were maintained under specific pathogen-free conditions at the Icahn School of Medicine at Mount Sinai. All animal experiments in this study were approved by the Institutional Animal Care and Use Committee of Icahn School of Medicine at Mount Sinai and were performed in accordance with the approved guidelines for animal experimentation.

### Diet and TAM administration

All mice were raised on Basal Diet 5053 in our facility. The diet was purchased from LabDiet (St. Louis, MO). Tamoxifen (TAM) (500 mg/kg) (Sigma, St Louis, MO) was added to Basal Diet 5053 to produce the 5053 TAM diet (TD.190129) and to Basal Diet 2019 to produce the 2019 TAM diet (TD.130968). The TAM was purchased from Envigo (Madison, WI).

### Food colorant treatment

Red 40 (Allura Red AC) was purchased from Sigma–Aldrich. Mice were exposed to Red 40 either in drinking water (0.025% w/v of Red 40, 0.25 g/L) or in the diet (containing 0.25 g/kg Red 40, diet 2019, TD.160647), as indicated.

### T-cell adoptive transfer

For CD4^+^ T-cell isolation, mLNs were digested in collagenase as described previously [11]. CD4^+^ T cells were enriched by positive immunoselection using CD4-(L3T4) microbeads (Miltenyi Biotec, Bergisch Gladbach, Germany). One million CD4^+^ T cells from the mLNs of *R23FR* mice in remission (Day 48) were enriched by using MACS beads and transferred into *Rag1*^*−*^^*/*−^ mice by intravenous (i.v.) injection.

### Flow cytometry and sorting

Cell suspensions from the *lamina propria* were prepared as described previously [11]. All cells were first preincubated with anti-mouse CD16/CD32 for blockade of Fc γ receptors and then washed and incubated for 30 min with the appropriate monoclonal antibody conjugates PE-Cyanine7-CD45 (Thermo Fisher Scientific, Cat# 25-0451-82; RRID: AB_469625), PE-CD3e (Thermo Fisher Scientific, Cat# 12-0031-82; RRID: AB_465496), APC-eFluor 780-CD4 (Thermo Fisher Scientific, Cat# 47-0042-82; RRID: AB_1272183) and APC-CD8a (Thermo Fisher Scientific, Cat# 17-0081-82; RRID: AB_469335) were used. 4,6-Diamidino-2-phenylindole (DAPI) (Invitrogen, Eugene, OR) was used to distinguish live cells from dead cells during cell analysis and sorting. DAPI^−^CD45^+^CD3^+^CD4^+^CD8^−^ T cells were purified with a BD influx cell sorter (BD Biosciences). Cells were >95% pure after sorting for single-cell RNA sequencing or ex vivo cytotoxicity assays.

### Single-cell RNA-sequencing (scRNA-seq)

The sorted cellular suspensions (DAPI^−^CD45^+^CD3^+^CD4^+^CD8^−^) were loaded on a 10× Genomics Chromium instrument to generate single-cell gel beads in emulsion (GEMs). Approximately 12,000 live CD3^+^CD4^+^CD8^−^ cells were loaded per channel (3 mice/group/channel). Single-cell RNA-Seq libraries were prepared using the following Single Cell 3′ Reagent Kits: a Chromium^™^ Single Cell 3′ Library & Gel Bead Kit v2 (PN-120237), a Single Cell 3′ Chip Kit v2 (PN-120236) and an i7 Multiplex Kit (PN-120262) (10× Genomics) following the Single Cell 3′ Reagent Kits v2 User Guide (Manual Part #CG00052 Rev A). Libraries with an estimated targeted cell recovery of 7000 cells were run on an Illumina HiSeq 4000 as 2 × 150 paired-end reads, one full lane per sample, for ~>73% sequencing saturation. All samples were processed in parallel during library preparation and sequenced on the same flow cell to minimize batch effects.

### Single-cell RNA-seq mapping

Cell Ranger Single Cell Software Suite, version 2.0.0, was used to perform sample demultiplexing, barcode and unique molecular identifier (UMI) processing, and single-cell 3′ gene counting. A detailed description of the pipeline and specific instructions can be found at https://support.10xgenomics.com/single-cell-gene-expression/software/pipelines/latest/what-is-cell-ranger. For cells from Diet 5053-treated mice, we recovered 6621 cells in total. The median number of genes per cell was 1583, the mean number of reads per cell was 53,436, and the median UMI count per cell was 4799. For cells from Diet 2019-treated mice, we recovered 6903 cells in total. The median number of genes per cell was 1763, the mean number of reads per cell was 52,216, and the median UMI count per cell was 5823.

### Clustering and further Seurat analysis functions

Initial clustering was performed using the R package ‘Seurat’ (Version 4.1.0) [56]. Cells with fewer than 800 genes and more than 4% of reads mapped to mitochondrial genes were excluded from the analysis. Genes with low expression (detected in fewer than three cells) were also removed. Doublets were predicted by the R package DoubletFinder and removed from the analysis independently. After filtration, we obtained 6388 cells for the Diet 2019-treated mice and 6220 cells for the Diet 5053-treated mice. Cell cycle stage annotation was performed using the CellCycleScoring function. For analysis of cells from Diet 2019-treated mice, the data were normalized using the NormalizeData function and scaled using the ScaleData function (the cell cycle scores, total UMI counts per cell and percentages of mitochondrial features were considered sources of unwanted variation and were regressed out). Principal component analysis (PCA) was performed using the top 2000 variable genes, and significant PCs were selected using the JackStraw function. Then, the FindNeighbors function was run using the top 30 dimensions. Subsequently, the FindClusters function was run to generate cell clusters (9 clusters with resolution = 0.5). UMAP dimensionality reduction was calculated on the top 30 PCs using the RunUMAP function. Cluster marker genes were identified using the FindAllMarkers function with the following additional setting: min.pct = 0.25, thresh.use = 0.25. KEGG pathway enrichment analysis of the upregulated genes in each cluster was performed with the package enrichR (https://cran.r-project.org/package=enrichR).

For comparison of cells from Diet 5053-treated mice and Diet 2019-treated mice, cells from the two groups were merged after filtering. The cell cycle scores together with the total UMI counts per cell, percentages of mitochondrial features and individual sample effects were considered sources of unwanted variation and were regressed out using the Seurat package [57]. Then, the data were processed and analyzed as described above. We obtained 11 clusters with PCA = 30 and resolution = 0.6.

### Primary colonic epithelial cell culture

C57BL/6 mouse primary colonic epithelial cells (Cell Biologics, Chicago, IL) were cultured in complete mouse epithelial cell medium (Cell Biologics, Chicago, IL) at 37 °C in a CO_2_ incubator in culture flasks or plates precoated with Gelatin-Based Coating Solution (Cell Biologics) according to the manufacturer’s instructions.

### Cytotoxicity assays against epithelial cells ex vivo

For ex vivo cytotoxicity assays, effector cells were isolated by flow sorting using the DAPI^−^CD45^+^CD3^+^CD4^+^CD8^−^ marker from pools of at least six large intestines of *R23FR* or *FR* mice. C57BL/6 mouse primary colonic epithelial cells were used as target cells. Effector (E) and target (T) cells were cocultured in medium in the presence of 10 ng/ml recombinant mouse IL-2 (PeproTech, Rocky Hill, NJ), 10 ng/ml recombinant mouse IL-7 (PeproTech) and 10 ng/ml recombinant mouse IL-15 (Peprotech) at 37 °C with 5% CO_2_ for the indicated hours at various E:T cell ratios. The experiments were performed at least in quadruplicate cultures.

### Cell death analysis by IncuCyte assay

IncuCyte analysis using integrated robust kinetic real-time high-content imaging with Annexin V labeling has been described previously [23, 24]. Briefly, kinetic experiments were performed with an IncuCyte ZOOM (Model 4459, Essen Bioscience, Ann Arbor, MI) residing in a tissue culture incubator. Experiments were conducted for 24 h with data collection every hour. Using the 10× objective, a single plane of view was collected per well for 96-well plate assays. Data were collected from the phase contrast and red channels (Ex: 565/05 nm; Em: 625/05 nm; acquisition time: 800 ms) were collected for all experiments. The images collected were 1392 × 1040 pixels at 1.22 μm/pixel. Automated image analysis was accomplished using ZOOM software (V2018A) and defined by images collected using the specific cell lines and fluorescent reporters pertaining to the experiment. The data are always expressed as the events per well. The images of cell culture were white-point-corrected to normalize visualization of the green and red pixel background in images lacking a significant fluorescent signal.

### Cell death analysis by flow cytometry

Cell death was analyzed by PI staining followed by flow cytometry. PI^+^Epcam^+^ cells were counted as dead cells for flow cytometric analysis.

### Cell death analysis by CellTiter-Glo luminescent cell viability assay

Cytotoxicity was also determined using a CellTiter-Glo Luminescent Cell Viability Assay Kit (Promega, Madison, WI) according to the manufacturer’s instructions. The percentage of survival was calculated as the (experimental luminescence − effector spontaneous luminescence)/(target spontaneous luminescence) × 100. In ex vivo blockade experiments, an anti-mFASL mAb (BioLegend, San Diego, CA; 40 μg/ml), anti-mTRAIL mAb (BioLegend; 20 μg/ml) and z-VAD-fmk (Sigma; 20 μM) were added to the indicated final concentration at the start of the cytotoxic assay. To evaluate the role of perforin in CD4^+^ CTL-mediated cytotoxicity, effector cells were pretreated with an inhibitor of perforin, SN34960 [27], at a concentration of 10 μM for 30 min before being incubated with the target cells in the presence of SN34960. Pretreated effector (E) and target (T) cells were cocultured for 24 h at the indicated E:T-cell ratio. Treatment with SN34960 at the concentration used in the study for the indicated time showed no toxic effects against effector and target cells as determined by cell survival. Cytotoxicity assays of the cells with antibodies or inhibitors were performed using a CellTiter-Glo Luminescent Cell Viability Assay Kit.

### Colonic epithelial cell enteroid culture and cell death assay

Mouse organoids were established and maintained at 37 °C as three-dimensional spheroid cultures in Matrigel (Corning, Bedford, MA) with L-WRN conditioned medium from isolated crypts collected from the intestines of C57BL/6 mice as previously described [58]. To maximize the early growth of developing primary spheroids from intestinal crypts, the tissue medium was supplemented with Y27632 (10 μM, Tocris Bioscience, Bristol, UK), an inhibitor of Rho-associated protein kinase (ROCK), and SB431542 (10 μM, Tocris Bioscience, Bristol, UK), an inhibitor of the transforming growth factor-β type I receptor, for 2–3 days [58]. For enteroid death assays, 20 enteroids and 80,000 CD4^+^ T cells were cocultured by resuspending them in Matrigel and plating them (5 μl per well in 100 μl of L-WRN-conditioned medium per well) in 96-well flat-bottom plates (Corning, Corning, NY). After 24 h, the viability of the enteroids was measured using a CellTiter-Glo Luminescent Cell Viability Assay Kit (Promega, Madison, WI) according to the manufacturer specifications, with the exception that 100 μl of reagent was added to 100 μl of culture for a final volume of 200 μl before reading.

### Transwell chamber assay

Primary IECs (10^4^/well) were seeded in a 96-well Transwell chamber (Corning No. 3381; 0.4 µm pore size, Kennebunk, ME) bottom receiver plate. Effector cells (CD4^+^ T cells) (4 × 10^4^/well) were plated in the top chamber insert. The transwell chamber was incubated at 37 °C for 24 h. The cytotoxicity of the cells in the bottom chamber was determined by using a CellTiter-Glo Luminescent Cell Viability Assay Kit.

### Hydrodynamic gene delivery of IL-23 minicircle DNA

Hydrodynamic gene delivery of IL-23 minicircle DNA into mice and determination of serum IL-23 after minicircle DNA injection were performed as described previously [12].

### FITC-dextran assay

Mice were kept without food and water for 4 h, and then FITC-dextran (#FD4-1G, Sigma) was administered by oral gavage at a concentration of 40 mg/ml in 400 μl (16 mg) per mouse (~800 mg/kg). Five hours later, plasma was isolated from peripheral blood, mixed 1:1 with PBS and analyzed on a plate reader at an excitation wavelength of 485 nm and an emission wavelength of 535 nm [59].

### In vivo antibody treatment

Blockade of IFN-γ and depletion of CD4^+^ cells were performed in an adoptive transfer model. Briefly, CD4^+^ T cells from *R23FR* mice in remission (Day 48) were adoptively transferred to *Rag1*^*−/*−^ mice. These recipient mice were fed Red 40 in drinking water (0.25 g/L) for 1 week and then switched back to normal water. One milligram of anti-IFN-γ (XMG1.2, BioXcell), 0.2 mg of anti-CD4 (GK1.5, BioXCell) or their isotype antibodies were administered by intraperitoneal injection at Day 13 and Day 14. Ab-injected mice were orally gavaged with Red 40 (1 mg/mouse) at Day 14. The mice were sacrificed 12 h after oral gavage of Red 40, and the large intestine was taken for histological analysis.

### Histology

Tissues were dissected, fixed in 10% phosphate-buffered formalin, and then processed into paraffin sections. Five-micrometer sections were stained with hematoxylin and eosin (H&E) for histological analyses. All the sections were evaluated for a wide variety of histological features, including epithelial integrity, the number of goblet (mucin-producing) cells, stromal inflammation, crypt abscesses, erosion, and submucosal edema. The severity of disease was then classified as described previously [11].

### Immunofluorescence staining

Immunofluorescence staining was performed as described previously [11, 12, 60, 61]. Briefly, tissues were dissected, fixed in 10% phosphate-buffered formalin, and then processed into paraffin sections. Five-micrometer sections were dewaxed by immersion in xylene (twice for 5 min each time) and hydrated by serial immersion in 100%, 90%, 80%, and 70% ethanol and PBS. Antigen retrieval was performed by microwaving the sections for 15 min in Target Retrieval Solution (DAKO, Carpinteria, CA). The sections were washed with PBS (twice for 10 min each time), blocking buffer (10% bovine serum albumin in Tris-buffered saline) was added, and the sections were incubated for 1 h. The sections were then incubated with primary antibodies in blocking buffer overnight at 4 °C. The antibodies used for immunofluorescence staining in this study were as follows: anti-CD3 (Abcam, ab11089), anti-Cleaved Caspase-3 (Cell Signaling Technology, 9661), anti-T-bet/TBX21 (Cell Signaling Technology, 97135), and anti-pan-keratin (Abcam, ab6401). After washing, conjugated secondary Abs were added, and the sections were incubated for 35 min. The slides were next washed and mounted with Fluoromount-G (Southern Biotech, Birmingham, AL). Images were captured using a Nikon fluorescence microscope and Nis-Elements BR imaging software. After setting the acquisition conditions according to the brightest sample, all the acquisition parameters were kept the same at all times, including the laser intensity, the exposure time, the gain of the photomultiplier, the offset of the histogram and the image magnification. On average, 15 determinations were made for each mouse examined. The Cleaved Caspase-3^+^ area was measured with ImageJ/FIJI [62].

### Isolation of IFN-γ^+^ and IFN-γ^−^ CD4^+^ T cells

For isolation of IFN-γ^+^ and IFN-γ^-^ CD4^+^ T cells from the *lamina propria*, lamina propria leukocytes from *R23FR* mouse ceca were incubated with a mouse IFN-γ catch reagent (Miltenyi Biotec, 130-090-516) for 5 min on ice. The cells were then incubated at a concentration of 1 × 10^5^ cells/ml in warmed medium at 37 °C under slow continuous rotation. After 45 min, IFN-γ was detected with a PE-anti-IFN-γ detection antibody (Miltenyi Biotec), and CD4^+^ T cells were stained with anti-CD45, anti-CD3e, anti-CD4 and anti-CD8a antibodies (Thermo Fisher Scientific). DAPI^–^CD45^+^CD3^+^CD8^−^CD4^+^IFN-γ^+^ and DAPI^−^CD45^+^CD3^+^CD8^−^CD4^+^IFN-γ^−^ cells were isolated by cell sorting (FACSDiva) for ex vitro killing assays and bulk RNA-seq.

### Bulk RNA-seq

Cells were directly sorted into TRIzol reagent (Invitrogen). Total RNA from purified cells was extracted using an RNeasy Micro Kit (Qiagen) according to the manufacturer’s instructions. The samples were shipped on dry ice to the Center for Functional Genomics and the Microarray & HT Sequencing Core Facility at the University at Albany (Rensselaer) for bulk RNA sequencing [62]. RNA quality was assessed using a NanoDrop (Thermo Scientific) and Bioanalyzer Total RNA Pico assay (Agilent). Total RNA with an RNA integrity number (RIN) value of 8 or greater was deemed of good quality to perform the subsequent protocols. One hundred micrograms of total RNA was oligo-dT-primed using a SMART-Seq v4 Ultra Low Input RNA Kit (Clontech), and the resulting cDNA was amplified using 15 cycles of PCR. The double-stranded cDNA (dscDNA) was purified using AMPure XP magnetic beads and assessed for quality using a Qubit dsDNA HS assay and an Agilent Bioanalyzer high-sensitivity dscDNA chip (expected size ~600 bp–9000 bp). An Illumina Nextera XT kit was used for library preparation, wherein 125 pg of dscDNA was fragmented and adaptor sequences were added to the ends of fragments. Next, 12 cycles of PCR amplification were performed. The DNA library was purified using AMPure XP magnetic beads, and the final library was assessed using a Qubit dsDNA HS assay for concentration and an Agilent Bioanalyzer high-sensitivity DNA assay for size (expected range ~600–740 bp). Library quantitation was also performed using a NEBNext Library Quant Kit for Illumina. Each library was then diluted to 4 nM, pooled and denatured as per standard Illumina protocols to generate a denatured 20 pM pool. Single-end 75 bp sequencing was performed on an Illumina NextSeq 500 by loading a 1.8 pM library with 5% PhiX into a 75-cycle high-output flow cell. The RNA-seq data were checked for quality using the Illumina FastQC algorithm on Basespace.

### Transcriptome analyses

RNA-Seq data were mapped to the mouse reference genome (UCSC/mm10) using TopHat version 2.1.0 [63]. Gene-level sequence counts were extracted for all annotated protein-coding genes using htseq-count version 0.6.1 [64] by taking the strict intersection between reads and the transcript models associated with each gene. Raw count data were filtered to remove weakly expressed genes with fewer than five counts in any sample. Differentially expressed genes between groups were analyzed using the Bioconductor EdgeR package version 3.10.2 Bioconductor/R [65, 66]. Statistically significant differentially expressed genes between groups (*Q* < 0.05) were selected in genewise log-likelihood ratio tests that were corrected for multiple testing by the Benjamini and Hochberg FDR values. Ingenuity Pathway Analysis (IPA) was used to annotate and visualize genes by function and pathway. GO analysis of significantly regulated genes was performed using ClueGO for GO terms containing at least three genes, redundancy was reduced using GO term fusion, connections were based on a kappa of 0.4, the top GO term was selected based on the %Genes/Term value, and GO terms with *Q* values < 0.05 (FDR) were considered significantly enriched.

### Quantification of fecal Lcn-2 by enzyme-linked immunosorbent assay (ELISA)

Freshly collected or frozen fecal samples were reconstituted in PBS containing 0.1% Tween 20 (100 mg/ml) and vortexed for 20 min to obtain homogenous fecal suspensions. These samples were then centrifuged for 10 min at 12,000 *rpm* and 4 °C. Clear supernatants were collected and stored at −20 °C until analysis. Lcn-2 levels were estimated in the supernatants using a Duoset murine Lcn-2 ELISA kit (R&D Systems, Minneapolis, MN). The concentration of Lcn-2 was normalized to the weight of the input feces.

### Statistical analysis

The data are shown as the mean values ± SEMs throughout. No statistical method was used to predetermine the sample size. Statistical analyses were performed with GraphPad Prism 7 software (GraphPad, La Jolla, CA). Differences between groups were analyzed with the nonparametric Mann–Whitney test. Differences were considered significant when *p* *<* 0.05, and levels of significance are specified throughout the figure legends.

## Supplementary information

Supplementary Figures
