# Supplementary material for: IFN-γ+ cytotoxic CD4+ T lymphocytes are involved in the pathogenesis of colitis induced by IL-23 and the food colorant Red 40
Source: Cell Mol Immunol. 2022 Apr 25;19(7):777–90. doi: 10.1038/s41423-022-00864-3 (PMC9243055; doi:10.1038/s41423-022-00864-3)
Supplement: Supplementary file 1 — Supplementary Figures [file 41423_2022_864_MOESM1_ESM.docx]

Supplementary Figures


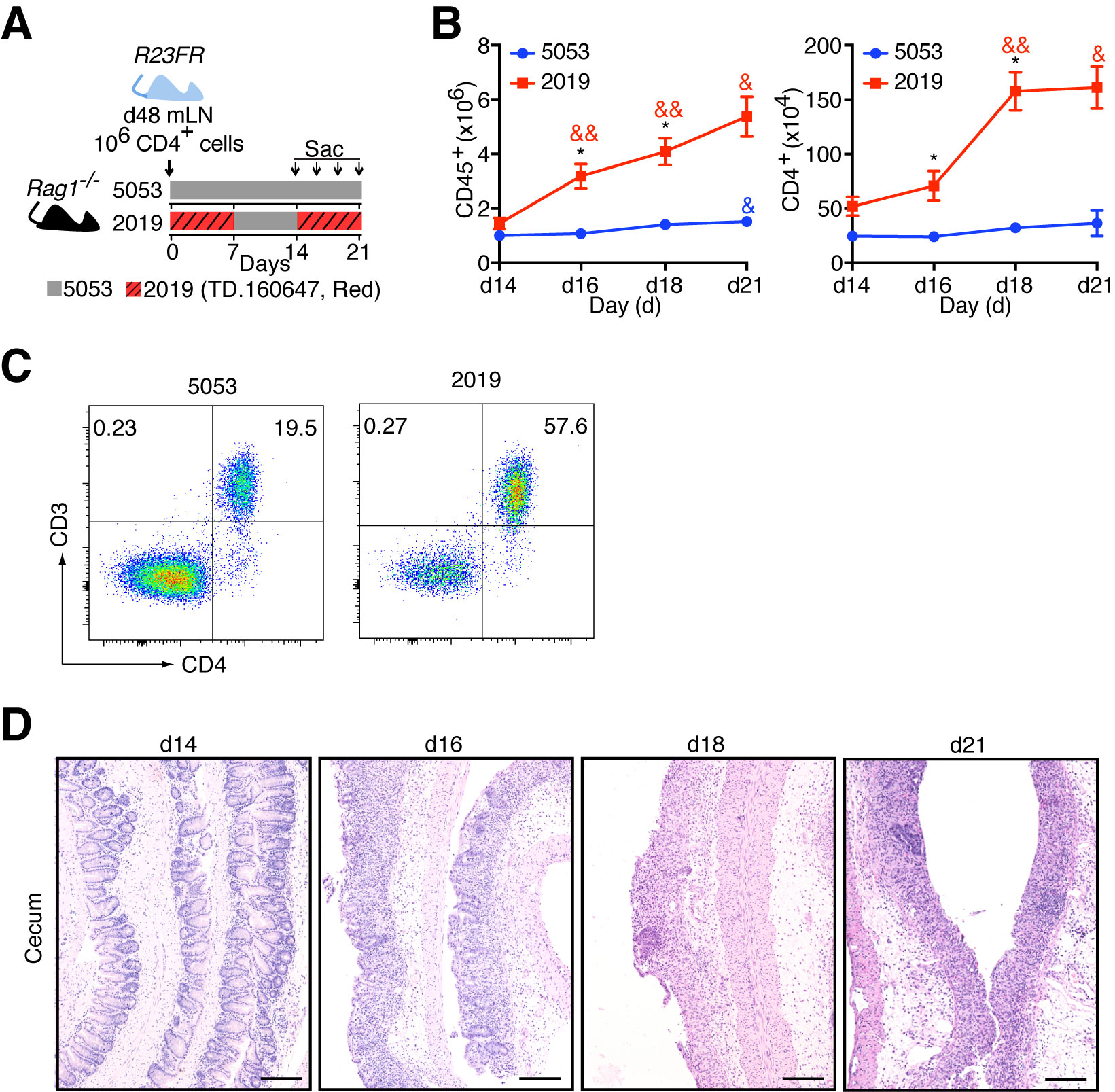


**Fig S1. Colitis development in adoptively transferred mice.**

(**A**) *R23FR* CD4^+^ T cell transfer *Rag1^-/-^* model. Schematic representation of the model. CD4^+^ T (10^6^ cells) were obtained from mLNs of *R23FR* mice during the remission phase (d48) and transferred into *Rag1^-/-^* recipient mice fed with different diets (diet 5053 or diet 2019). (**B**) The number of CD45^+^ (left) and CD4^+^ (right) cells in the cecum of adoptively transferred *Rag1^-/-^* mice at different time points. Asterisks (*) indicate a statistically significant difference in the number of cells in the cecum of mice treated with diet 2019 compared with number of cells in the cecum of mice treated with diet 5053. Ampersands (^&^) indicate a statistically significant difference in the number of cells compared with number of cells at d14 within each group. Data are means ± SEM. * p<0.05, ^&^ p< 0.05, ^&&^ p< 0.01, by nonparametric Mann-Whitney test. (n=3-5 per group per time point). (**C**) The representative flow cytometry plots of blood in adoptively transferred *Rag1^-/-^* mice gated on CD45^+^ cells. Notice that all CD3^+^ cells are CD4^+^ T cells. (**D**) Representative H&E-stained cecum sections of *Rag1^-/-^* mice adoptively transferred with *R23FR* CD4^+^ T cells and fed with diet 2019 at different time points (n=5 per time point). Scale bars, 100 μm.

**
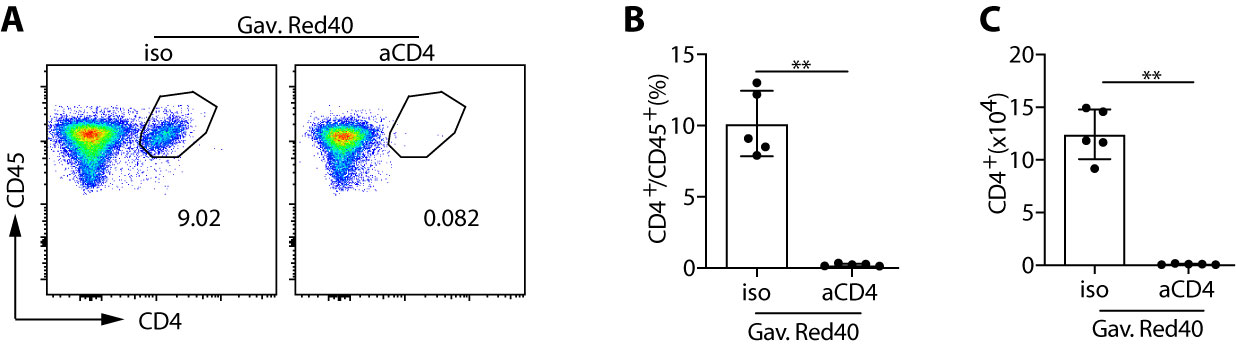
**

**Fig. S2 Depletion of CD4^+^ lymphocytes after anti-CD4 injection.**

(**A**) Representative flow cytometry plots gated on live cells. (**B** and **C**) Relative (**B**) and absolute (**C**) number of CD4^+^ cells in the cecum of mice injected with isotype and anti-CD4 antibody. Each dot represents one mouse. Data are means ± SEM. **p<0.01.


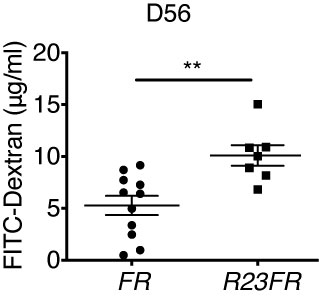


**Fig. S3 Intestinal permeability in colitic R23FR mice.**

Intestinal permeability in R23FR mice and control FR mice at d56 after TAM + Red 40 treatment assessed by measuring serum FITC-Dextran levels 5h after administration. Graphs show means ± SEM, n= 6-11 mice/group. ** p<0.01, by nonparametric Mann-Whitney test.

**
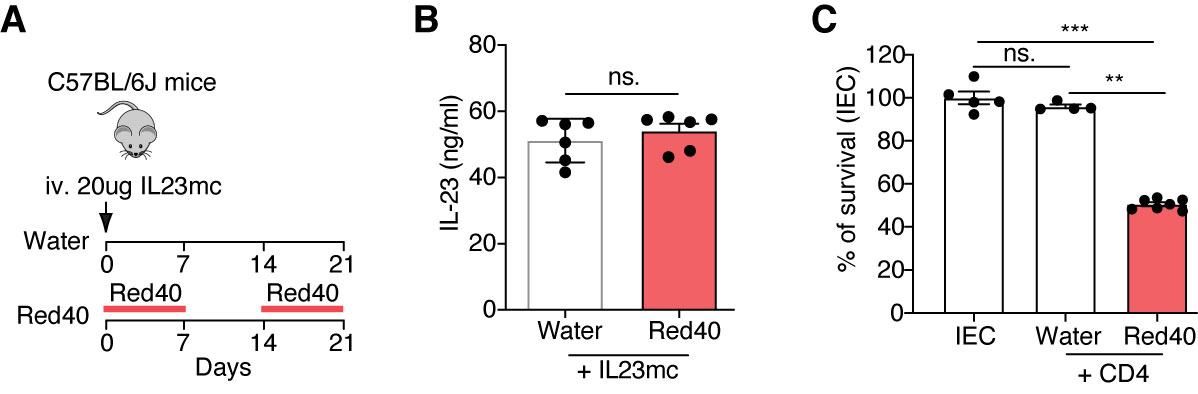
**

**Fig. S4 Red 40 treatment of wild-type mice with dysregulated expression of IL-23 results in induction of cytolytic CD4^+^ T cells.**

(**A**) Schematic representation of the experiment. 20 μg mouse IL-23 minicircle DNA (IL23mc) was injected through the tail vein per mouse into C57BL/6J mice. The mice were treated with or without 0.25 g/L Red 40 in drinking water after injection. Flow-sorted CD4^+^ T cells from large intestine at day 21 were used as effector cells. (**B**) Serum IL-23 levels at day 21 after mcDNA injection. n= 6/group. (**C**) Survival of target intestinal epithelial cells after co-culture with CD4^+^ T cells was assessed with the CellTiter-Glo Luminescent Cell Viability Assay kit (ratio of effector: target=4:1). n=4-7. Each dot represents one well/condition from a representative of two independent experiments with similar results. Ns, not significant, *p<0.05, ***p<0.001, by nonparametric Mann-Whitney test.

**
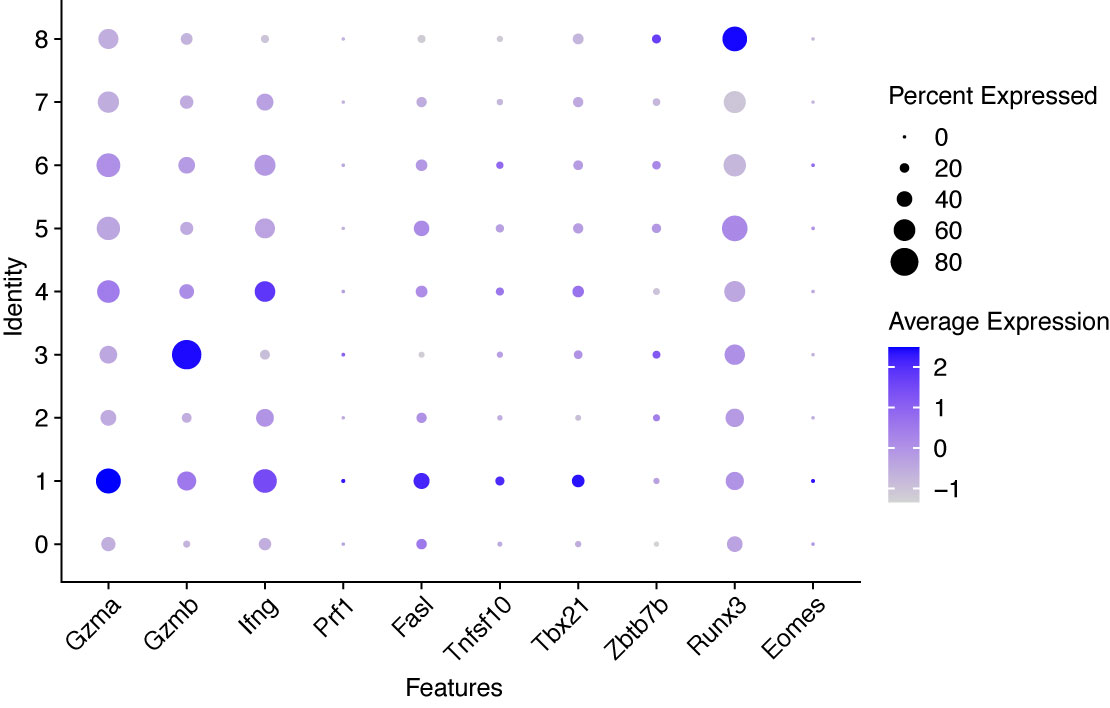
**

**Fig. S5 Single-cell expression pattern of cytotoxicity-related genes and transcription factors.**

Dot plots show the single-cell expression pattern of cytotoxicity-related genes and transcription factors in the clusters of the colitogenic CD4^+^ T cells from adoptively transferred *Rag1^-/-^* mice. The size of the dot corresponds to the percentage of cells expressing the marker in each cluster. The color represents the average expression level.

**
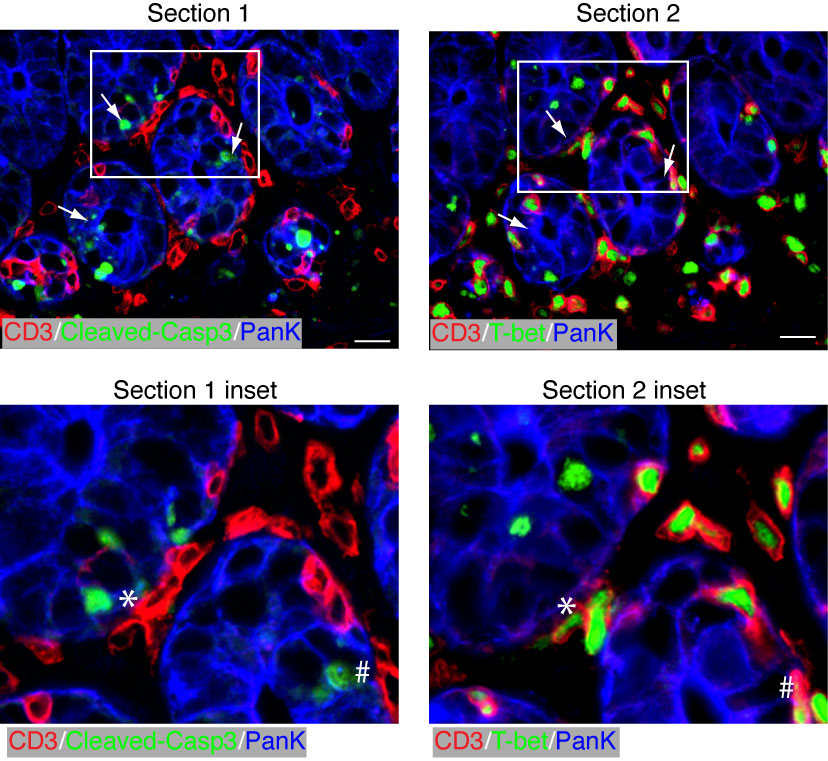
**

**Fig. S6 Immunostaining of cecal T-bet^+^ T cells in adoptively transferred mice with colitis.**

Adjacent tissue sections (sections 1 and 2) were used to visualize T-bet^+^ (green, left panel), T cells (CD3^+^) (Red), and apoptotic (Cleaved Caspase-3^+^) (green, right panel) epithelial cells (Pan-keratin^+^)(blue) in the cecum of Red-40-gavaged adoptively transferred *Rag1^-/-^* mice. The bottom panels show a higher-magnification image of the insets in the upper panels. Arrowheads indicate that the T cells that were close proximity to the cleaved Caspase-3^+^ epithelial cells were T-bet^+^. * and # in the bottom indicate the same cell/area between adjacent sections. Scale bars =100 μm.
